# Acute Coronary Syndrome Remodels the Protein Cargo and Functions of High-Density Lipoprotein Subfractions

**DOI:** 10.1371/journal.pone.0094264

**Published:** 2014-04-15

**Authors:** Ying Tan, Ting Rong Liu, Shui Wang Hu, Di Tian, Chen Li, Jian Kai Zhong, Hai Ge Sun, Tian Tian Luo, Wen Yan Lai, Zhi-Gang Guo

**Affiliations:** 1 Division of Cardiology, Nanfang Hospital, Southern Medical University, Guangzhou, Guangdong, P.R. China; 2 Laboratory of Pathophysiology, Southern Medical University, Guangzhou, Guangdong, P.R. China; National Institute of Environmental Health Sciences, United States of America

## Abstract

**Objectives:**

This study examined alterations in the functions and proteome of high-density lipoprotein (HDL) subfractions (HDL2 and HDL3) isolated from patients with acute coronary syndrome (ACS) compared with control subjects.

**Methods:**

We measured HDL subfraction cholesterol efflux capacity, inflammatory index (HII), paraoxonase-1 (PON1) activity, and lipid hydroperoxide (LOOH) levels in both male age-matched controls and the ACS group (n = 40/group). Additionally, proteomic analysis was used to monitor changes in the HDL subfraction proteome between controls and ACS subjects.

**Results:**

Both HDL2 and HDL3 from ACS patients had greater HII and LOOH levels compared with controls (*P<*0.001); PON1 activity and cholesterol efflux capacity in both HDL2 and HDL3 from the ACS group were significantly less than those of controls (*P<*0.001). Using proteomic analysis, we demonstrated that, compared with the control group, nine proteins were selectively enriched in HDL3 from subjects with ACS, and ras-related protein Rab-7b was decreased in HDL3. Additionally, in the ACS subjects, 12 proteins were decreased in HDL2 and 4 proteins were increased in HDL2.

**Conclusions:**

Functional HDL subfractions shifted to dysfunctional HDL subfractions during ACS, and the functional impairment was linked to remodeled protein cargo in HDL subfractions from ACS patients.

## Introduction

A strong inverse correlation between high-density lipoprotein cholesterol (HDL-C) level and coronary artery disease (CAD) has fostered intensive research seeking to target HDL metabolism for therapeutic gain [1], [2]. HDL is thought to protect against atherosclerosis by promoting reverse cholesterol transport (RCT), potentially through anti-oxidative and anti-inflammatory capacities [3], [4]. However, it appears that the relationship between HDL levels and CAD risk is more complex and not just merely related to the plasma HDL-C levels. In recent years, studies have confirmed that quantifying HDL-C concentration alone provides limited information about HDL's cardioprotective effects [5]. Reports of marked heterogeneity in the particle composition and biological properties of HDL have reinforced a need to assess HDL function [6]. Recent studies have suggested that the ability of HDL to promote RCT from macrophages correlates with atherosclerosis independent of HDL-C [4]. Consequently, the assessment of HDL functions has become a novel target to investigate the association between HDL and CAD risk.

Notably, it has been found that HDL particles may become dysfunctional or even proinflammatory in chronic and inflammatory diseases [7]. Acute coronary syndrome (ACS) constitutes a unique inflammatory milieu. For example, the proinflammatory cytokine CXCL16 is more highly expressed in subjects with acute myocardial infarction than in those with chronic atherosclerosis [8]. Under such inflammatory conditions, it has been shown that the protein and phospholipid moieties of HDL are substantially altered, thereby modifying the functional characteristics of the HDL particles [9]. Indeed, animal studies have convincingly demonstrated that changes in proteins involved in HDL metabolism can promote atherosclerosis, even when plasma levels of HDL-cholesterol are elevated [10]. A few studies, mostly on a small scale, have suggested that patients with underlying inflammatory conditions tend to have HDL that has a decreased anti-inflammatory capacity [11]–[14].

More recent studies have suggested that the HDL proteome is implicated in HDL functionality, identification of HDL-associated proteins involved in lipid metabolism, complement activation, acute-phase response protein, and proteolysis regulation [15], [16]. These data indicate the value of identifying differences in the HDL proteome between different populations. However, present studies regarding HDL proteomics mainly focus on the HDL level. Human plasma consists of two main subfractions: HDL2 and HDL3. The different HDL subfractions have different biological functions and play different roles in the efficiency of RCT. The functional significance of these different HDL subfractions is not quite clear. Proteomic studies of HDL subfractions have also rarely been reported.

In this study, a comparative proteomic analysis and functional assessment of HDL subfractions have been performed between ACS and control patients. Our study demonstrated that functional HDL subfractions shifted to dysfunctional HDL subfractions during ACS and that the functional impairment was linked to remodeled protein cargo in HDL subfractions from ACS patients. Furthermore, this study is the first to reveal that both HDL2 and HDL3 have significantly elevated serum amyloid P-component (SAP) and decreased ras-related protein Rab-7b levels in ACS patients. In addition, we also confirmed that quantification of HDL-C concentration alone provides limited information regarding HDL's cardioprotective effect.

## Materials and Methods

### Subject selection

The protocol for blood sampling for this study was approved by the Research Ethics Board of NanFang Hospital affiliated with Southern Medical University in Guangzhou, China, and all subjects provided written informed consent to participate in this study. Blood anticoagulated with EDTA was collected after an overnight fast from 40 subjects with established ACS (ACS group, 27 males and 13 females) and from 40 apparently healthy people (control group, 27 males and 13 females). Plasma samples were immediately isolated, thoroughly mixed with sucrose (final concentration of 0.5%) and EDTA (1 mg/mL) to prevent HDL oxidation and aggregation, and frozen at −80°C until analysis.

The diagnosis of ACS was confirmed by clinical assessment by a cardiologist, including symptoms consistent with angina, any vessel with 50% occlusion in the setting of elevated cardiac enzymes (creatinine kinase, creatinine kinase -MB, troponin I, or troponin T), and/or dynamic electrocardiogram changes. Exclusion criteria included uncontrolled hypertension, triglycerides≥5 mmol/L, severe obesity (body mass index≥30 kg/m^2^), alcohol intake>14 drinks/week, and the presence of thyroid, hepatic, or renal disease, or diabetes. Additionally, subjects were excluded if they had autoimmune disease, or any chronic or acute infectious or inflammatory illness. None of the ACS subjects received lipid-lowering medications for at least 6 weeks before blood collection. The control subjects had no known history of CAD, were not hyperlipidemic, and had no family history of premature CAD. Data were collected on all subjects, as indicated in Table 1. The investigation conforms to the principles outlined in the Declaration of Helsinki.

**Table 1:** Comparison of basic clinical data between the ACS and control groups.

Parameter	Control group	ACS group
Age (years)	49.20±5.04	51.50±6.27
Cholesterol (mmol/L)	4.94±0.47	5.12±0.72
Triglycerides (mmol/L)	1.10±0.33	1.16±0.39
LDL-C (mmol/L)	2.96±0.42	3.32±0.65^*^
HDL-C (mmol/L)	1.28±0.25	1.22±0.25
hsCRP (log mg/L)	1.86±0.39	2.79±0.51^*^

Values are means±SD. *P<0.01 compared with the control group. ACS, acute coronary syndrome; LDL-C, low-density lipoprotein cholesterol; HDL-C, high-density lipoprotein cholesterol; hsCRP, high-sensitivity C-reactive protein. Data are presented here after log transformation of hsCRP levels (for nonparametric results).

### Isolation of HDL subfractions

HDL2 and HDL3 were isolated by a two-step discontinuous density-gradient ultracentrifugation method [17]. Each plasma sample (4 mL) was adjusted to a density of 1.24 g/mL with KBr (0.3816 g/mL) and was added to a 8.9 mL centrifuge tube (Beckman); then, it was slowly overlaid with KBr/phosphate buffer solution (0.0834 g/mL, d = 1.063 g/mL). The samples were centrifuged at 58100 rpm (290000 *g*) for 4 h at 15°C in a Beckman L-80 XP equipped with a Ti 90 rotor (fixed angle; Beckman Instruments, Fullerton, CA, USA), and HDL subfractions (1.063<d<1.21 g/mL) located in the middle of the tube were then collected separately by penetrating the tube with a syringe. Subsequently, both HDL fractions were further purified by a second ultracentrifugation that was performed under the same conditions as described above but for 2 h after KBr/phosphate solution (d = 1.24 g/mL) was added to the two fractions. Finally, HDL2 and HDL3 were collected from the top of the tube, desalted with desalting buffer (NH_4_ HCO_3_, 12 mM, pH 7.1) and PD-10 columns (Sephadex G-25 M, Amersham Bioscience), and either directly used or stored at −70°C for further analysis. Final protein concentrations were determined by a BCA protein assay.

### Assay of HDL-mediated cholesterol efflux from macrophages

HDL-mediated cholesterol efflux from macrophages was measured as previously described [4]. J774 macrophages (American Type Culture Collection) were incubated with Dulbecco's modified Eagle's medium (DMEM) containing 0.2% bovine serum albumin (BSA), 50 mg/ml oxidized low density lipoprotein (ox-LDL), and 2 µCi/ml ^3^H-cholesterol (Perkin-Elmer) for 24 h. The cells were then washed and incubated in DMEM containing 0.2% BSA and 0.3 mM 8-Br-cAMP (Sigma) to yield ABCA1-enriched cells. After another wash with serum-free medium, the cells were incubated for the next 4 h with 0.2% BSA in DMEM in the presence of the HDL subfractions (HDL2 and HDL3; 50 µg/ml). Subsequently, the incubation medium was collected and filtered through a 0.45 mm glass fiber filter to remove cellular debris before counting the radioactivity. The cell monolayers were washed with phosphate-buffered saline and lysed with 1 ml of 0.1 M NaOH.

The radioactivity of the medium and cell lysates was measured by liquid scintillation spectrometry. Percent efflux was calculated by the following formula: [(microcuries of ^3^H-cholesterol in media containing HDL subfractions − microcuries of ^3^H-cholesterol in HDL subfraction-free media) ÷ total ^3^H-cholesterol radioactivity (cell lysates plus media)] ×100.

### Cell-free assay (CFA)

The CFA was described previously [18] using PEIPC as a fluorescence-inducing agent with some modifications. Briefly, DCFH-DA was dissolved in fresh methanol to 2.0 mg/mL and incubated at room temperature in the dark for 30 min for the release of DCFH. Ten microliters of PEIPC solution at 50 µg/mL and 90 µL of HDL subfractions containing dextran sulfate supernatant with 10 µg/mL cholesterol were mixed and divided into aliquots into flat-bottom, black polystyrene microtiter plates. The plates were then incubated at 37°C on a rotator for 1 h. Ten microliters of DCFH solution (0.2 mg/mL) was then added to each well, mixed, and incubated at 37°C with rotation for an additional 2 h. Fluorescence was determined with a plate reader (Spectra Max, Gemini XS; Molecular Devices) at excitation, emission, and cutoff wavelengths of 485 nm, 530 nm, and 515 nm, respectively, with the photomultiplier sensitivity set at medium. Values in the absence of HDL subfractions were normalized to 1.0; values>1.0 after the addition of the test HDL subfractions indicated proinflammatory HDL subfractions, while values<1.0 indicated antiinflammatory HDL subfractions.

### Fluorescence labeling

A total of 50 µg of each type of HDL subfraction sample was labeled respectively with CyDye 3 (control group) and CyDye 5 (ACS group). An internal standard (50 µg) comprised of equal amount of proteins from all samples (control and ACS group) was labeled with CyDye 2 and included in all gels. CyDyes were dissolved in anhydrous dimethylformamide and mixed with the samples (HDL subfractions) at a ratio of 400 pmol of CyDye to 50 µg of protein. The reaction was performed on ice and in the dark for 30 min and then terminated by adding 1 µl of 10 mM lysine under the same conditions for 10 min. Cy2, Cy3, and Cy5 images appeared blue, green, and red using ImageQuant TL software.

### 2-D gel electrophoresis and 2-D difference gel electrophoresis (DIGE) analysis

2D-DIGE was performed using IPGphor and Multiphor from Amersham Biosciences as described previously [19]. After the labeled protein samples were loaded per 24 cm, pH 3–10 NL immobilized pH gradient (IPG) strip, the first dimension electrophoresis was performed by using an Ettan™ IPGPhor focusing apparatus. Before the second dimension electrophoresis, IPG strips were equilibrated successively with dithiothreitol (DTT) and iodoacetamine buffers, each time for 15 min. Subsequently, proteins were separated by electrophoresis on 12.5% polyacrylamide Tris-glycine gels using an Ettan™ DALT Six System. Lights were avoided during the whole procedure.

Then, the gels were scanned with a Typhoon Imager 9400 scanner. Differential in-gel analysis and data quantification were performed using DeCyder V6.5 analysis software. All Cy2 images were set to internal standard images, and the glue containing most of protein points was set as the reference glue. Finally, glues with differential spots matching to the corresponding differential spots in the preparation glue were excavated with Ettan™ Spot picker and stored in a 1.5-ml Eppendorf tube at −20°C for further mass spectrometry (MS) analysis.

### MS analysis and protein identification

The MS analysis was performed using the method described by Karlsson et al. [17]. Peptides obtained after tryptic digestion were mixed 1∶1 with matrix containing 5 mg/mL of α-cyano-4-hydroxycinnamic acid (CHCA, Sigma, USA) and then spotted on a target plate of a MALDI linear reflectron mass spectrometer. Analysis of peptide masses was performed using an ABI 4800 MALDI-TOF/TOF MS (Applied Biosystems, USA). Peptide mass fingerprinting (PMF) obtained through MS was retrieved using Mascot software, while MS and MS/MS combined patterns were searched using NCBInr and Swissprot databases to identify proteins. The human species database was used, with a molecular weight range and quality error toleration set at 800–4000 Da and 50 PPM, respectively. Moreover, it was required that more than four peptides matched and the protein score was greater than 95% for highly credible results.

### Western blotting analysis

Aliquots of each sample (HDL subfractions) were separated by SDS-PAGE, transferred to polyvinylidene difluoride (PVDF) membranes, and probed with polyclonal rabbit anti-haptoglobin antibody (Abcam Inc, USA), polyclonal rabbit anti-SAP antibody (Epitomics, USA), and anti-hemopexin antibody (Abcam Inc, USA).

### Other procedures

Serum triglycerides (TG), total cholesterol (TC), LDL-cholesterol (LDL-C), and HDL-C concentrations were measured with an automated biochemical analyzer (Type AU5421, Olympus, Japan). High-sensitivity C-reactive protein (hs-CRP) concentrations were determined by nephelometry (Behring Nephelometer 100 Analyzer, Germany). HDL subfraction paraoxonase activity were measured as previously described [20]–[21]. The SAP concentration was determined by ELISA.

An independent sample t-test was used for analyzing the differences in variables between the ACS and control groups. SPSS 13.0 software was used for statistical analysis, and a statistically significant difference was considered at P<0.05.

## Results

### Clinical characteristics of study subjects

The clinical characteristics of the ACS group and the data for 40 age- and sex-matched healthy controls (control group) are shown in Table 1. Patients with ACS had significantly higher levels of LDL-C and hs-CRP relative to controls (P<0.01). However, there were no significant differences in TG, TC, and HDL-C levels (P>0.05) between the ACS and control groups. Subjects with ACS had a higher hs-CRP level, which may be related to their unique inflammatory milieu.

### Assessment of HDL and HDL subfraction functions

**Figure 1:**
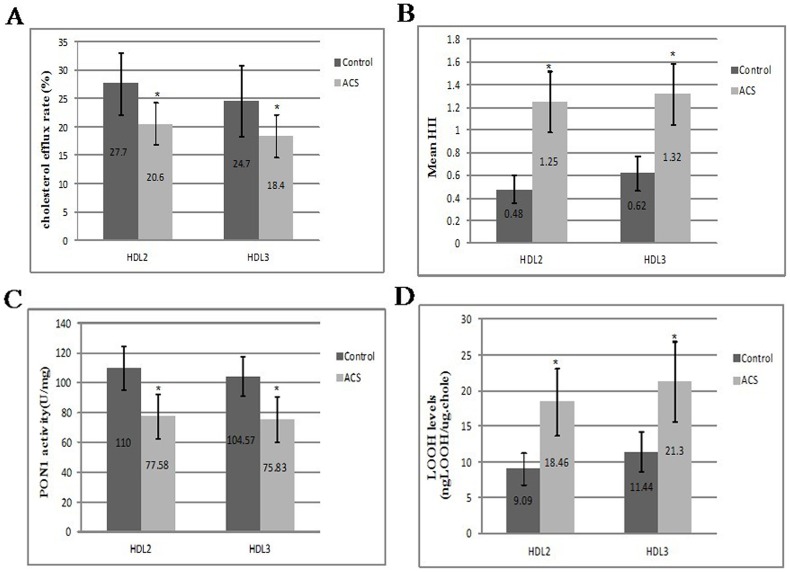
Comparison of HDL subfraction functions between acute coronary syndrome and control groups. The high-density lipoprotein subfraction cholesterol efflux capacity, inflammatory index (HII), paraoxonase-1 (PON1) activity, and LOOH levels were measured in both male age-matched controls and ACS subjects (n = 40/group). (A), HDL subfractions, HDL2 and HDL3, mediated reverse cholesterol transport capacity; (B), anti-inflammatory capacity; (C) and (D) anti-oxidant capacity. PON1 activity was normalized to mg of protein; *P<0.001 compared with the control group.

We measured the HDL and HDL subclass-mediated cholesterol efflux rate from macrophages, inflammatory index (HII), paraoxonase-1 (PON1) activity, and LOOH levels in both ACS subjects and controls. The results are shown in Table 2 and Figure 1. Compared with the control group, the cholesterol efflux rates in HDL, HDL2, and HDL3 from the ACS group were decreased by 28.4%, 25.6%, and 25.5%, respectively (P<0.001, Table 2 and Figure 1A); and the PON1 activities in HDL, HDL2, and HDL3 were also decreased by 24.3%, 29.5%, and 27.5% compared with the controls, respectively (P<0.001, Table 2 and Figure 1C). Meanwhile, the HII in HDL, HDL2, and HDL3 from the ACS group were increased 2.3 times, 2.6 times, and 2.1 times compared with the control group, respectively (P<0.001, Table 2 and Figure 1B); and the LOOH levels in HDL, HDL2, and HDL3 were increased 1.85 times, 2 times, and 1.9 times compared with the control group, respectively (P<0.001, Table 2 and Figure 1D).

**Table 2:** Comparison of HDL functions between the ACS and control groups.

Parameter	Control group	ACS group
PON1 Activity (U/mg)	93.33±23.29	70.65±14.43^*^
HII	0.56±0.17	1.28±0.14^*^
Cholesterol Efflux(%)	26.15±2.29	18.72±1.88^*^
LOOH(ng/ug.chole)	10.58±2.46	19.59±3.06^*^

Values are means±SD. *P<0.001 compared with the control group. ACS, acute coronary syndrome; PON1 activity was normalized to mg protein; HII, HDL inflammatory index.

### Proteomics results

Proteomic analysis revealed that differentially expressed HDL subfractions between the ACS and control groups were present on 48 spots. By MS, we demonstrated that compared with controls, HDL3 from the ACS group was selectively enriched in nine proteins (apolipoprotein A-I, apolipoprotein A-IV, apolipoprotein E, apolipoprotein L1, paraoxonase, alpha-1B-glycoprotein, SAP, vitamin D-binding protein, and fibrinogen gamma chain). Meanwhile, ras-related protein Rab-7b was decreased in HDL3. Additionally, 12 proteins were lower in HDL2 from ACS subjects (apoA-I, apoE, PON, apoA-IV, apoL1, haptoglobin, hemopexin, serotransferrin, complement factor B, ras-related protein Rab-7b, fibrinogen gamma chain, and Ig gamma-1 chain C region). Furthermore, four proteins were increased in HDL2 (SAP, alpha-1-antitrypsin, acid ceramidase, and vitamin D-binding protein).

**Figure 2:**
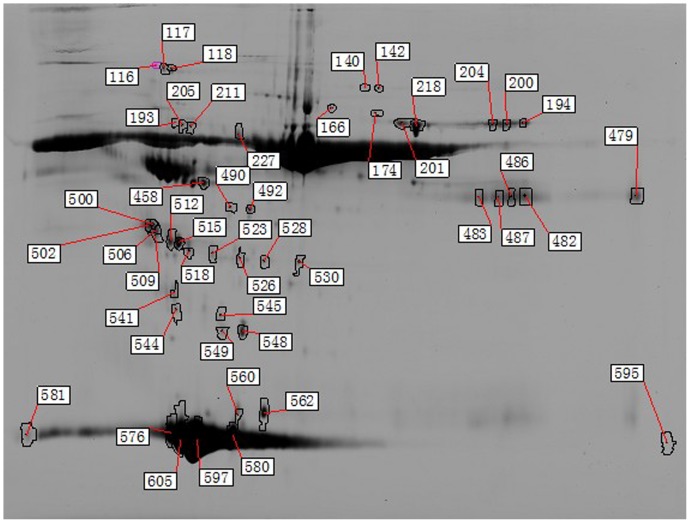
Forty-eight differentially expressed protein spots in HDL subfractions between the ACS group and controls analyzed by DeCyder 2D software. Proteins were extracted as described and separated in pH–10 NL IPG strips for the first dimension and 12.5% polyacrylamide for the second dimension. The image was acquired on a Typhoon 9400 scanner at 633/670 nm excitation/emission wavelengths. Spots detected by the analysis software are indicated.

**Figure 3:**
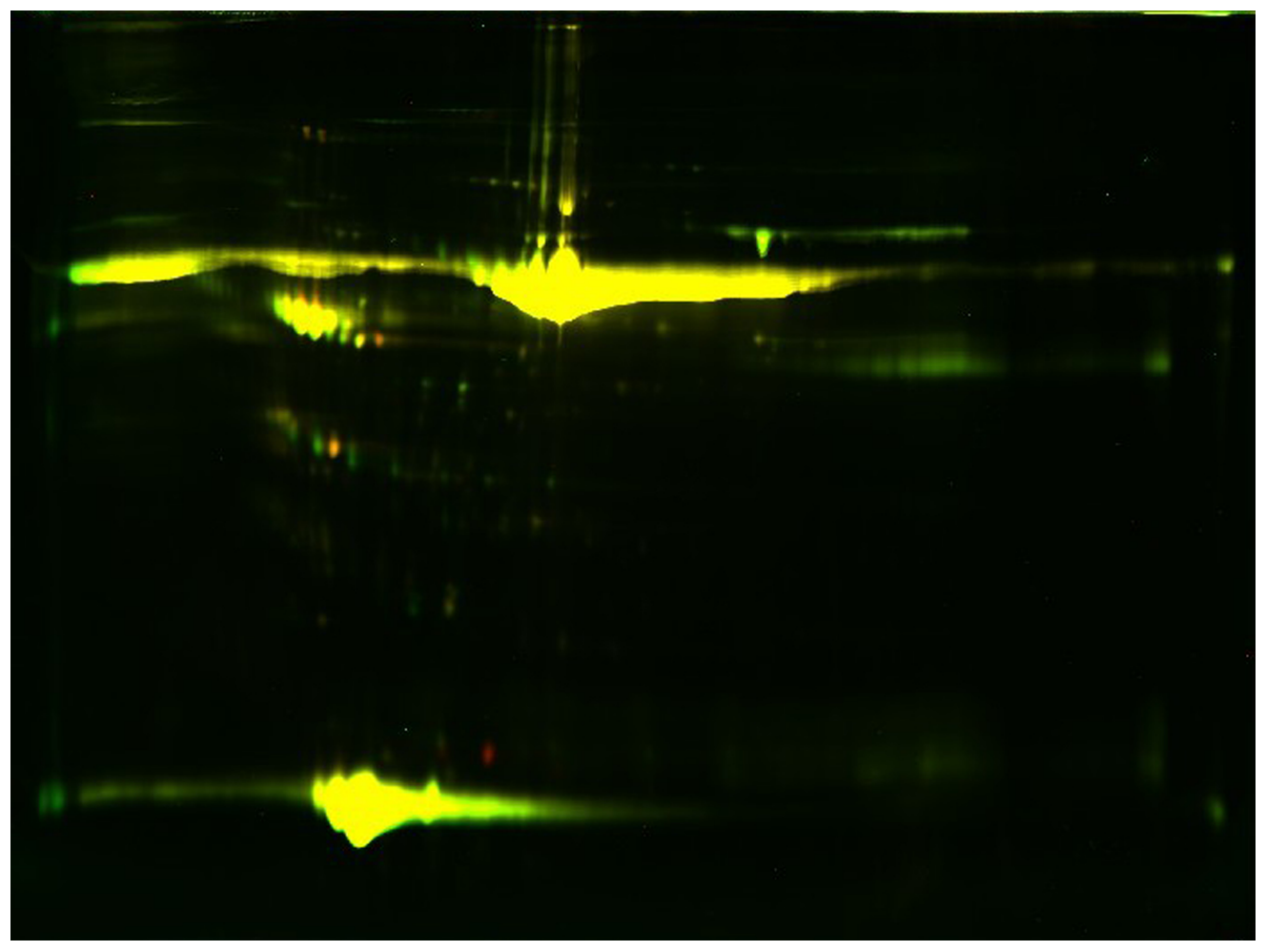
Overlapped sections of the 2D-DIGE proteome map of the acute coronary syndrome group and controls in HDL2. A total of 50 µg of each type of HDL subfraction sample was labeled with CyDye 3 (control group) and CyDye 5 (ACS group), respectively. An internal standard (50 µg) comprised of an equal amount of proteins from all samples (control and ACS group) was labeled with CyDye 2 and included in all gels. Cy2, Cy3, and Cy5 images appeared as blue, green, and red using the ImageQuant TL software. The green spots indicate downregulated proteins, while the red spots indicate upregulated proteins. Compared with the controls, 12 proteins were downregulated in HDL2 from ACS subjects, while 4 proteins were upregulated.

**Figure 4:**
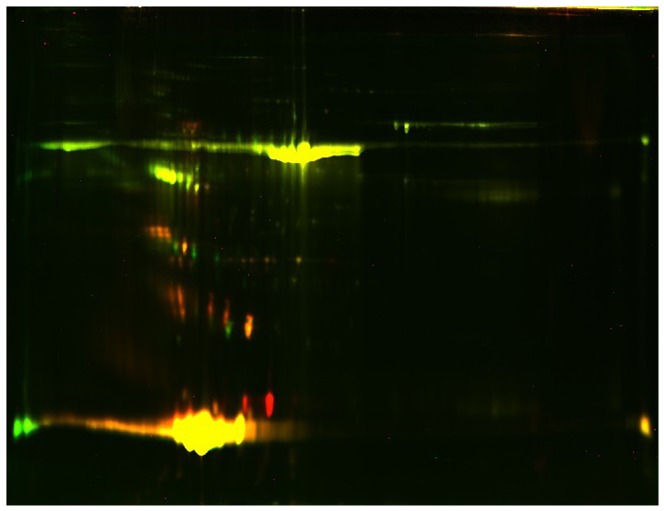
Overlapped sections of the 2D-DIGE proteome map of the acute coronary syndrome group and controls in HDL3. The green spots indicate downregulated proteins, while the red spots indicate upregulated proteins. Compared with the controls, nine proteins were selectively enriched in HDL3 from ACS subjects, while ras-related protein Rab-7b was decreased in HDL3.

The MS identification results are presented in Tables 3 and 4, and a 2D image is shown in Figure 2. In addition, a section of the gel labeled with the DIGE dyes is presented in Figures 3 and 4.

**Table 3:** Identification results of differentially expressed HDL2-associated proteins in the ACS group versus controls by mass spectrometry analysis.

SwissProt ID	Protein name	Spot	Ratio	Protein Score	Peptidic species Count	MW (kDa)	PI
Q13510	Acid ceramidase	117	1.63	57	7	45.09	7.52
P01009	Alpha-1-antitrypsin	118	1.64	88	8	46.89	5.37
P00751	Complement factorB	140	−1.62	106	10	86.85	6.67
		142	−1.63	127	12	86.85	6.67
P02787	Serotransferrin	174	−1.83	61	10	79.28	6.81
		201	−1.62	99	13	79.28	6.81
		204	−1.63	272	22	79.28	6.81
		218	−1.66	806	32	79.28	6.81
P02790	Hemopexin	227	−1.52	417	16	52.38	6.55
P02774	Vitamin D-binding protein	458	1.73	581	19	54.53	5.40
P01857	Ig gamma-1 chain C region	479	−1.73	287	12	36.60	8.46
		486	−1.58	123	10	36.60	8.46
P02679	Fibrinogen gamma chain	490	−1.94	299	16	52.11	5.37
		492	−1.68	380	15	52.11	5.37
P27169	Serum paraoxonase	509	−2.09	86	6	39.90	5.08
P06727	Apolipoprotein A-IV	512	−1.98	233	15	45.37	5.28
		515	1.74	382	19	45.37	5.28
P00738	Haptoglobin	518	−5.60	184	8	45.86	6.13
Q96AH8	Ras-related protein Rab-7b	523	−2.70	84	14	59.05	5.09
O14791	ApolipoproteinL1	528	−1.80	143	9	44.00	5.60
		530	−1.93	105	8	44.00	5.60
P02649	Apolipoprotein E	549	−7.41	350	18	36.25	5.65
P02647	Apolipoprotein A-I	562	6.07	501	24	30.76	5.56
		580	−1.50	459	21	30.76	5.56
		581	−6.29	421	23	30.76	5.56
		595	−1.89	210	19	30.76	5.56
P02743	Serum amyloid P-component	560	1.93	69	4	25.49	6.10

ACS denotes acute coronary syndrome. Ratio denotes a ratio of HDL3-associated proteins from the ACS group to HDL3-associated proteins from controls. MW denotes molecular weight. pI denotes isoelectric point.

**Table 4:** Identification results of differentially expressed HDL3-associated proteins in the ACS group versus controls by mass spectrometry analysis.

SwissProt ID	Protein name	Spot	Ratio	Protein Score	Peptidic species Count	MW (kDa)	PI
P04217	Alpha-1B-glycoprotein	193	1.58	145	14	54.81	5.58
		205	1.59	327	17	54.81	5.58
		211	1.96	545	19	54.81	5.58
P02774	Vitamin D-binding protein	458	1.97	581	19	54.53	5.40
P02679	Fibrinogen gamma chain	490	1.79	299	16	52.11	5.37
		492	1.91	380	15	52.11	5.37
P27169	Serum paraoxonase	500	3.67	81	5	39.90	5.08
		502	3.55	242	9	39.90	5.08
		506	4.66	108	10	39.90	5.08
		509	2.18	86	6	39.90	5.08
P06727	Apolipoprotein A-IV	515	3.52	382	19	45.37	5.28
Q96AH8	Ras-related protein Rab-7b	523	−2.35	84	14	59.05	5.09
O14791	ApolipoproteinL1	528	2.55	143	9	44.00	5.60
		530	1.88	105	8	44.00	5.60
P02647	Apolipoprotein A-I	541	4.87	134	12	30.76	5.56
		544	4.64	107	10	30.76	5.56
		562	62.85	501	24	30.76	5.56
		576	4.16	431	18	30.76	5.56
		580	2.36	459	21	30.76	5.56
		581	-2.56	421	23	30.76	5.56
		595	2.09	210	19	30.76	5.56
		597	2.87	345	16	30.76	5.56
		605	3.69	483	22	30.76	5.56
P02743	Serum amyloid P-component	560	15.34	69	4	25.49	6.10
P02649	Apolipoprotein E	545	6.22	378	20	36.25	5.65
		548	4.32	503	20	36.25	5.65

ACS denotes acute coronary syndrome. Ratio denotes a ratio of HDL3-associated proteins from the ACS group to HDL3-associated proteins from controls. MW denotes molecular weight. pI denotes isoelectric point.

### Validation of differential proteins by western blot and ELISA

**Figure 5:**
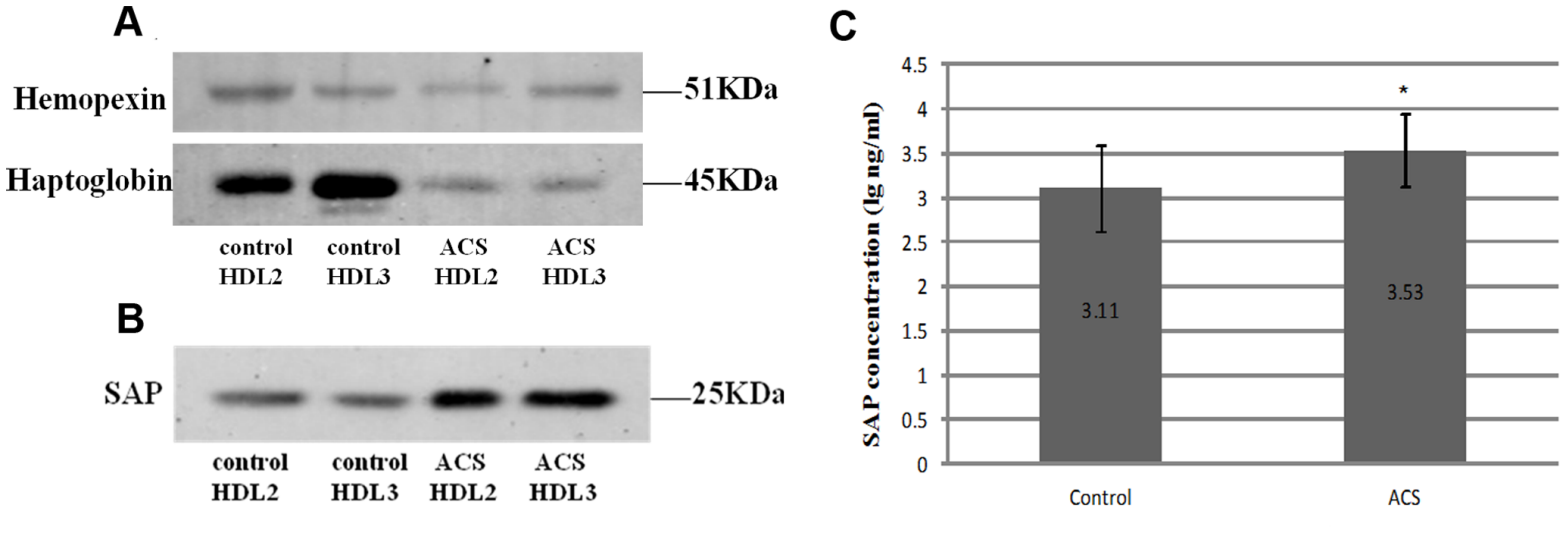
Validation of HDL-associated proteins by western blot and ELISA. (A) and (B), to confirm the results obtained by MS+MS/MS, HDL subfractions isolated from ACS patients and control subjects were subjected to immunoblot analysis. HDL subfraction-associated proteins were separated by SDS-PAGE, transferred to PVDF membranes, and probed using specific antibodies. Molecular mass is indicated on the right. (C), comparison of serum amyloid P-component concentrations between acute coronary syndrome and control groups. SAP denotes serum amyloid P-component. *P<0.001 compared with controls. Data are presented here after log transformation of SAP levels (for nonparametric results).

To validate the ability of our proteomic approach to identify proteins, we validated the three significant proteins (haptoglobin, hemopexin, and SAP) by western blot. We also measured the concentration of SAP by ELISA (Figure 5). The results showed that patients with ACS had higher SAP levels [(3.53±0.41) log ng/ml] compared with controls (3.11±0.48)log ng/ml, P<0.001].

## Discussion

Consistent with previous similar studies of HDL functions [4], [7], [19], we found that HDL particles from ACS patients were pro-inflammatory and had a reduced cholesterol efflux capability (Table 2). Importantly, we assessed the function of HDL subfractions (HDL2 and HDL3) and compared the HDL subfraction proteomes for the first time. Our study demonstrated that HDL subfractions from patients with ACS shifted to dysfunctional HDL subfractions, which reduced RCT as well as anti-inflammatory and anti-oxidant capacities (Figure 1). Meanwhile, the protein cargo was remodeled in ACS patients (Tables 3 and 4). Notably, there was no significant difference in HDL-C levels between the ACS and control groups (Table 1), suggesting that measurement of HDL-C concentrations, the current accepted clinical standard, does not provide adequate functional information to define HDL particles. To the best of our knowledge, our findings demonstrated for the first time that the HDL subfraction functions in ACS decreased, suggesting that ACS may impair the function of HDL subfractions and implicating the possibility that dysfunctional HDL contributes to accelerated atherosclerosis in ACS patients.

The role of inflammation in ACS is well established. Subjects with ACS had elevated hs-CRP levels (Table 1), which confirmed the inflammatory status of these subjects. It has been suggested that such inflammatory conditions are capable of remodeling the protein structure and composition of HDL [9], and even slightly altered diets can produce different HDL molecules [22]. For example, in vitro studies have demonstrated that oxidative damage impairs the ability of apolipoprotein A-I (apoA-I), the major HDL protein, to remove cholesterol from macrophages [23]. Our results suggest that ACS could impair HDL subfraction functions by triggering an inflammatory response, which is consistent with previous reports [24]. ACS radically changes the circulating environment for HDL and is involved in both acute inflammation and oxidative stress, due to pathogens or resulting from the ensuing damage [25].

To verify whether the protein components of HDL subfractions are altered during ACS, we compared the HDL subfraction proteomes of the control and ACS groups by proteomic analysis. Forty-eight different spots were found in HDL subfractions between the ACS and control groups (Figure 2). A total of 17 differentially expressed HDL-associated proteins were identified using MS (Tables 3 and 4). These proteins were related to lipid metabolism (apoA-I, apoA-IV, apoE, and apoL1), oxidative stress (PON1), inflammatory response (haptoglobin, hemopexin, ras-related protein Rab-7b, alpha-1B-glycoprotein, SAP, serotransferrin, fibrinogen gamma chain, and alpha-1-antitrypsin), and the immune system (complement factorB and Ig gamma-1 chain C region). Of those identified, the majority of the proteins (15/17) were consistent with well-established HDL-associated proteins and 5 proteins have well-characterized roles in lipid metabolism. In addition, the antioxidant properties of HDL were detected, which validates our experimental approach.

The majority of the proteins found to be differentially expressed are reported to have a role in cardiovascular diseases. Consistent with previous studies of HDL proteomics [6], [24], we found that the HDL3 from subjects with ACS increased selectively in four proteins (apoA-I, apoA-IV, apoE, and PON1) that play critical roles in lipid metabolism, macrophage biology, and oxidative stress (Table 3). Interestingly, the opposite results were observed in HDL2 (the less dense subfraction of HDL) from ACS subjects (Table 3). Three proteins (apoA-I, apoA-IV, and PON1) are well-known to possess anti-oxidative and anti-atherosclerotic properties [26]. Many lines of evidence indicate that apoE plays a role in modulating atherogenesis by promoting cholesterol efflux from macrophages [27]. The levels of these proteins in HDL3 markedly increased but were largely reduced in HDL2, supporting the proposal that conditions associated with ACS may promote the production of proteins in HDL3 by the liver or remodel HDL particles so that these proteins redistribute to denser HDL subfractions (i.e., HDL3) [28].

Haptoglobin (Hp) and Hemopexin (Hx) are plasma proteins with the highest binding affinity for Hb and heme, respectively. HDL-associated hemoglobin has been suggested to be a major mediator of the cellular effects of inflammatory HDL [29]. HDL from coronary heart disease (CHD) patients has been shown to contain significantly more Hb and its scavenger proteins, Hp and Hx [29]. Furthermore, Watanabe et al. have demonstrated that the association of Hb, Hp, and Hx proteins with HDL positively correlates with the inflammatory properties of HDL and systemic inflammation in CHD patients [29]. In contrast, we observed a significant decrease in HDL2-associated Hp and Hx levels in ACS subjects (Table 3), which may be attributable to differences among subjects. In contrast with CHD patients, the total hemoglobin levels in hospitalized ACS subjects are reduced by approximately 1.5 g/dL versus baseline [30], [31], which may subsequently make its scavenger protein (Hp and Hx) levels reduced. Moreover, previous studies [29] were based on HDL, but HDL2 as the less dense subfraction of HDL has a unique protein cargo differing from HDL. Therefore, we believe that this may not necessarily be a result of proinflammatory HDL-remodeling, and further studies are required to elucidate the role of Hp and Hx in ACS. Nagy et al. [32] found that the inhibition of heme release from globin by haptoglobin and sequestration of heme by hemopexin suppress hemoglobin-mediated oxidation of lipids of atheromatous lesions and attenuate endothelial cytotoxicity, indicating that the decrease of Hp and Hx in HDL2 obtained from ACS subjects may be an important factor causing functional HDL to shift to dysfunctional HDL.

In addition, we also found that alpha-1-antitrypsin (A1AT) levels in HDL2 were elevated in ACS subjects compared with controls (Table 3). A recent study has demonstrated that HDL-associated A1AT as a serine protease inhibitor was able to inhibit extracellular matrix degradation and apoptosis induced by elastase in human vascular smooth muscle cells (VSMCs) and in mammary artery cultured ex vivo, which is a new potential antiatherogenic property of HDL [33]. Thus, we speculate that A1AT, as an acute-phase protein, may be relatively elevated in HDL2 during ACS in order to exert an antiatherogenic effect.

A major finding of this study was that both HDL2 and HDL3 possess significantly elevated SAP levels and decreased ras-related protein Rab7b levels in ACS subjects compared with controls (Tables 3 and 4). This is the first report that SAP and ras-related protein Rab-7b reside in HDL subfractions. SAP is a main member of the pentraxin family and shares 70% homology with CRP. Studies indicate that SAP does not exist in normal aortic intima but exists in human atherosclerotic aortic intima and that plasma SAP levels are positively associated with cardiovascular disease in the elderly [30], [34]. Consistent with previous reports [30], [34], a significant increase in serum SAP levels was observed in ACS patients relative to controls (Figure 5C). These findings indicate that SAP may serve as a marker of atherosclerotic development and/or progression underlying angina and MI. Furthermore, our study revealed that HDL subfractions from ACS patients possess significantly elevated SAP levels (15.34 times higher than controls in HDL3), suggesting that SAP may have vital effects on HDL subfraction functions. Song et al. have demonstrated that SAP associated with HDL promotes SR-BI-dependent cholesterol efflux and that lipid-free SAP enhances ABCA1-dependent cholesterol efflux [35]. Additionally, SAP binding to amyloid-like structures in oxidized LDL blocks macrophage uptake of modified LDL, serving to prevent atherosclerosis [36]. It will be necessary to further explore the roles of SAP in lipid metabolism and atherosclerosis.

Rab7b is a recently identified member of the Rab GTPase protein family and promotes lysosomal degradation of TLR4 and TLR9. Studies have shown that Rab7b can negatively regulate TLR4 and TLR9 signaling by promoting lysosomal degradation of TLR4 and TLR9 and subsequently impairing activation of MAPK and NF-kB pathways, reducing inflammatory factor production in macrophages [37]. These findings indicate that the decrease in Rab7b associated with HDL subfractions during ACS may impair HDL subfraction functions and contribute to accelerated atherosclerosis in ACS patients.

Furthermore, our proteomic study showed that vitamin D binding protein (VDBP) was significantly increased in HDL subfractions from ACS patients (Tables 3 and 4). VDBP is related to the immune system because it can bind to the C5 complement components and selective deglycosylation of VDBP transforms VDBP into a macrophage-activating factor (VDBP-MAF) in response to vascular injury [38]. The upregulation of this protein associated with HDL subfractions could be indicative of proinflammatory and proatherogenic functions.

In summary, we report here for the first time that functional HDL subfractions shifted to dysfunctional HDL subfractions during ACS, and the functional impairment was linked to remodeled protein cargo in HDL subfractions during ACS. These differentially expressed HDL-associated proteins might be useful indicators of cardiovascular risk. Future work on the proteins identified should be performed to determine their effects on HDL subfraction functions.
